# Epigenetic state and gene expression remain stable after CRISPR/Cas‐mediated chromosomal inversions

**DOI:** 10.1111/nph.20403

**Published:** 2025-01-29

**Authors:** Solmaz Khosravi, Rebecca Hinrichs, Michelle Rönspies, Reza Haghi, Holger Puchta, Andreas Houben

**Affiliations:** ^1^ Leibniz Institute of Plant Genetics and Crop Plant Research Gatersleben Corrensstrasse 3 06466 Seeland Germany; ^2^ Joseph Gottlieb Kölreuter Institute for Plant Sciences – Molecular Biology Karlsruhe Institute of Technology Fritz‐Haber‐Weg 4 76131 Karlsruhe Germany

**Keywords:** chromosome engineering, CRISPR/Cas, epigenetics, gene expression, inversion

## Abstract

- The epigenetic state of chromatin, gene activity and chromosomal positions are interrelated in plants. In *Arabidopsis thaliana*, chromosome arms are DNA‐hypomethylated and enriched with the euchromatin‐specific histone mark H3K4me3, while pericentromeric regions are DNA‐hypermethylated and enriched with the heterochromatin‐specific mark H3K9me2. We aimed to investigate how the chromosomal location affects epigenetic stability and gene expression by chromosome engineering.
- Two chromosomal inversions of different sizes were induced using CRISPR/Cas9 to move heterochromatic, pericentric sequences into euchromatic regions. The epigenetic status of these lines was investigated using whole‐genome bisulfite sequencing and chromatin immunoprecipitation. Gene expression changes following the induction of the chromosomal inversions were studied via transcriptome analysis.
- Both inversions had a minimal impact on the global distribution of histone marks and DNA methylation patterns, although minor epigenetic changes were observed across the genome. Notably, the inverted chromosomal regions and their borders retained their original epigenetic profiles. Gene expression analysis showed that only 0.5–1% of genes were differentially expressed genome‐wide following the induction of the inversions.
- CRISPR/Cas‐induced chromosomal inversions minimally affect epigenetic landscape and gene expression, preserving their profiles in subsequent generations.

## Introduction

There is a general correlation between the chromosomal position of a DNA sequence, the epigenetic state of the chromatin as well as gene activity (Grewal & Moazed, 2003; Liu *et al*., 2016). Chromosome arms are euchromatin‐enriched, whereas centromeric and pericentromeric regions are heterochromatic in many species (Roudier *et al*., 2009). Euchromatin, which is the decondensed fraction of chromatin, contains mostly active genes (Strahl *et al*., 1999). By contrast, heterochromatin, the condensed chromatin fraction, is poor in genes and gene activity (Fischer *et al*., 2006; Liu *et al*., 2016). The formation and maintenance of the chromatin status is regulated epigenetically by DNA methylation and post‐translational histone modifications. Heterochromatin is enriched in hypermethylated DNA and dimethylated histone H3K9 (H3K9me2) (Soppe *et al*., 2002). By contrast, euchromatin is linked with trimethylated H3K4 (H3K4me3) and less C‐methylation of DNA.

Position effect variegation (PEV), discovered in the fruit fly *Drosophila melanogaster* (Gowen & Gay, 1934) and humans (Finelli *et al*., 2012), as well as the telomere position effect (TPE), discovered in budding yeast, are examples for possible effects of the chromosomal position on gene expression (Gottschling *et al*., 1990). Genes undergo differential expression in PEV because chromosomal inversions create new heterochromatin–euchromatin borders, and euchromatic genes juxtaposed to heterochromatic regions undergo heterochromatin‐induced gene silencing (Hessler, 1958; Elgin & Reuter, 2013). The impact of the chromosomal position on gene expression is well‐studied in the case of the expression of the 45S rDNA loci in *Arabidopsis thaliana* (Mohannath *et al*., 2016). Also, other studies suggest that changes in gene expression follow the introduction of chromosomal rearrangements, such as inversions or translocations, due to reorganization of large regulatory domains (Naseeb *et al*., 2016). They are also reported to cause the modification of genetic regions adjacent to the breakpoints (Lavington & Kern, 2017), the epigenetic environment of translocated and adjacent regions (Wesley & Eanes, 1994; Fournier *et al*., 2010), or to cause nuclear reorganization (Fournier *et al*., 2010; Harewood *et al*., 2010). However, it is unknown whether the reported gene expression and epigenetic changes occurred immediately after the introduction of the chromosomal rearrangements or whether they were established over time in subsequent generations.

To unravel the effect of chromosomal inversions on the epigenetic state of chromatin and the activity of genes in *A. thaliana*, we employed CRISPR/Cas‐assisted chromosome engineering for the generation of two differently sized chromosomal inversions (Rönspies *et al*., 2022a). The inversions were first confirmed by sequencing of the inversion junctions and fluorescent *in situ* hybridization (FISH). Then, the epigenetic state of these lines was compared with wild‐type (WT) plants with the help of whole‐genome bisulfite sequencing (WGBS) and chromatin immune precipitation followed by sequencing (ChIP‐seq) using antibodies recognizing H3K4me3 and H3K9me2 as eu‐ and heterochromatic histone marks, respectively. Finally, the effect of the chromosomal rearrangements on the activity of genes was analyzed. Our results showed that none of the studied inverted chromosome segments and their neighboring regions changed in epigenetic marks and gene expression besides minor genome‐wide effects, demonstrating the robustness of the epigenome and transcriptome following CRISPR/Cas‐induced chromosomal restructuring, at least in the following generations.

## Materials and Methods

### Generation of CRISPR/Cas9‐induced *A. thaliana* inversion lines

#### Cloning of T‐DNA constructs

The Gateway‐compatible plasmids pEn‐Sa‐Chimera and pDe‐Sa‐Cas9, containing *Staphylococcus aureus* Cas9 under the control of an egg cell‐specific promotor (pDe‐Sa‐Cas9 EC), were used for cloning of the transfer DNA (T‐DNA) constructs (Katzen, 2007; Steinert *et al*., 2015; Schmidt *et al*., 2020). The spacer sequences were integrated into individual pEn‐Sa‐Chimera vectors as annealed oligonucleotides via *Bbs*I restriction digestion. The used spacer sequences that are specific for both borders of the inversions are listed in Supporting Information Table S1. The first guide RNA (gRNA) cassette was integrated into pDe‐Sa‐Cas9 EC through a classical cloning approach by *Mlu*I restriction digestion and ligation. The second gRNA cassette was transferred into the vector via a Gateway LR reaction.

#### Plant cultivation and transformation

For the transformation of the *A. thaliana* Col‐0 plants with the CRISPR/Cas expression constructs, 4‐ to 5‐wk‐old plants were transformed via *Agrobacterium tumefaciens*‐mediated floral dip transformation (Clough & Bent, 1998). After transformation, the plants were cultivated for 4–5 wk until seed maturity. To generate sterile plant cultures, seeds were surface‐sterilized with 4% sodium hypochlorite and stratified overnight at 4°C. Stratified seeds were sown on Murashige & Skoog (MS) medium (10 g l^−1^ saccharose, pH 5.7 and 7.6 g l^−1^ plant agar) containing gentamicin (0.075 g l^−1^) and cefotaxime (0.5 g l^−1^) to select transgenic plants. The selected transgenic plants (T1) were either used for TIDE analysis to determine the efficiency of Cas9 or transferred to the greenhouse and cultivated until seed set to obtain T2 seeds for further experiments.

#### Extraction of genomic DNA and TIDE analysis

To determine the efficiency of Cas9 in inducing targeted double‐strand breaks in the target regions, plants were transformed with expression constructs containing the spacer sequence and the Cas enzyme under the control of a ubiquitin promoter. In T1 leaf material, the mutation rate was analyzed by TIDE analysis, which was used as a proxy to determine the cutting efficiency (Brinkman *et al*., 2014). The DNA of 10 primary transformants and a Col‐0 WT control of *A. thaliana* (L.) Heynh. was extracted, and the targeted region amplified via PCR. Primers were designed in a way that they were located *c*. 350 bp upstream and downstream of the predicted cleavage site. The primers are listed in Table S2. The reaction mixture was purified using the peqGOLD Cycle‐Pure kit (VWR International) and subjected to Sanger sequencing by Eurofins Genomics. Using the TIDE online tool (https://tide.nki.nl/) with default settings, the mutation rate was calculated as a proxy for the cutting efficiency in each sample. The mean value of the individual samples was calculated to determine the cutting efficiency of each target site.

#### Establishment of homozygous inversion lines

The harvested T2 seeds were stratified and sown on germination medium without additives, and the plates were cultivated in a growth chamber at 22°C under 16 h : 8 h, light : dark conditions for 2 wk. Afterwards, 40 plants per T2 line were used for bulk DNA extraction. A PCR was performed on the T2 pools to screen for the presence of the inversions using inversion junction‐specific primers (Table S2). If a T2 pool tested positive for the respective inversion, the DNA of the individual plants of this line was analyzed separately by a PCR to identify individual plants carrying the desired restructuring. To verify the induced inversions, the junctions were subjected to Sanger sequencing by Eurofins Genomics, and the results were analyzed by sequence alignment using the software ApE (v.2.0.55). Plants that were found to carry the inversion were propagated in the glasshouse for 6–7 wk until seed set. For genotyping in the T3 generation, PCRs were performed using specific primers for the WT and inversion junctions (Table S2). Additionally, the T3 lines were tested for Mendelian segregation using a chi‐squared test with the critical value χ^2^ (1; 0.95) on the genotyping results.

### Cytogenetic analysis

#### Chromosome spread preparation

Closed flower buds of *c*. 1 mm length were harvested and fixed in freshly prepared Carnoy's fixative solution (3 : 1 v/v, ethanol: glacial acetic acid) for 48 h at room temperature (RT). Chromosome spreads were prepared from fixed buds according to Mandáková & Lysak (2016), with the minor change of reducing the enzyme digestion time to 60 min. Prepared slides were washed with 70% ethanol for 2 min, followed with 2× SSC for 1 min. Then, they were postfixed in 4% formaldehyde in 2× SSC for 10 min. Next, slides were washed twice in 2× SSC for 5 min and finally dehydrated in an ethanol gradient (70%, 90% and 100%, each step 2 min). The slides were air‐dried for at least 1 h and counterstained with DAPI (2 μg ml^−1^ in Vectashield). Finally, the slides were analyzed by fluorescence microscopy and the ones containing many pachytene chromosomes were selected for FISH.

#### Fluorescence *in situ* hybridization

Single‐copy oligo FISH probes were designed using the Abor Biosciences' Co. proprietary software (Han *et al*., 2015). Regions of *c*. 100 kb region were selected upstream or downstream of the CRISPR/Cas9‐induced break points, and 45 bp long single‐copy sequences were used for the probe design. Nonoverlapping target‐specific oligonucleotides were synthesized as myTags libraries (Arbor Bioscience, Ann Arbor, MI, USA). The pAL1, containing a 180 bp repeat (Martinez‐Zapater *et al*., 1986), was labeled using Atto647N using the nick‐translation labeling kit (Jena Biosciences, Jena, Germany).

For performing the FISH experiments, the procedure described by Kubalová *et al*. (2023) was followed except for the following changes. The selected myTags probes were pooled in a microtube and placed in a SpeedVac concentrator (Eppendorf) for evaporation. Afterwards, the probes were reconstituted in 1.5 μl of ddH_2_O. Per slide, 1400 ng per myTags probe was used. Before adding the myTags probe, 75 ng centromere‐specific probe was added to 18.5 μl of hybridization mixture (50% formamide; 10% dextran sulfate; 10% salmon sperm DNA; and 2× SSC) per slide and denatured at 95°C for 10 min, then placed on ice for 5 min. Next, the reconstituted myTags probes were added to the mixture and the mixture was added to the slides. Slides were incubated for 20 min at 37°C in a wet chamber and then denatured on a hot plate (70°C) for 3 min. Finally, slides were hybridized for 48 h at 37°C. Posthybridization washing was carried out by washing in 2× SSC at 42°C for 20 min under shaking conditions. Micrographs were captured using an epifluorescence microscope (Olympus BX61) equipped with a cooled charge‐coupled device (CCD) camera (Orca ER; Hamamatsu Photonics, Hamamatsu, Japan) and pseudo‐colored by the Adobe Photoshop 6.0 software.

#### Plant growth conditions for RNA‐seq and epigenome analysis

**Fig. 1:**
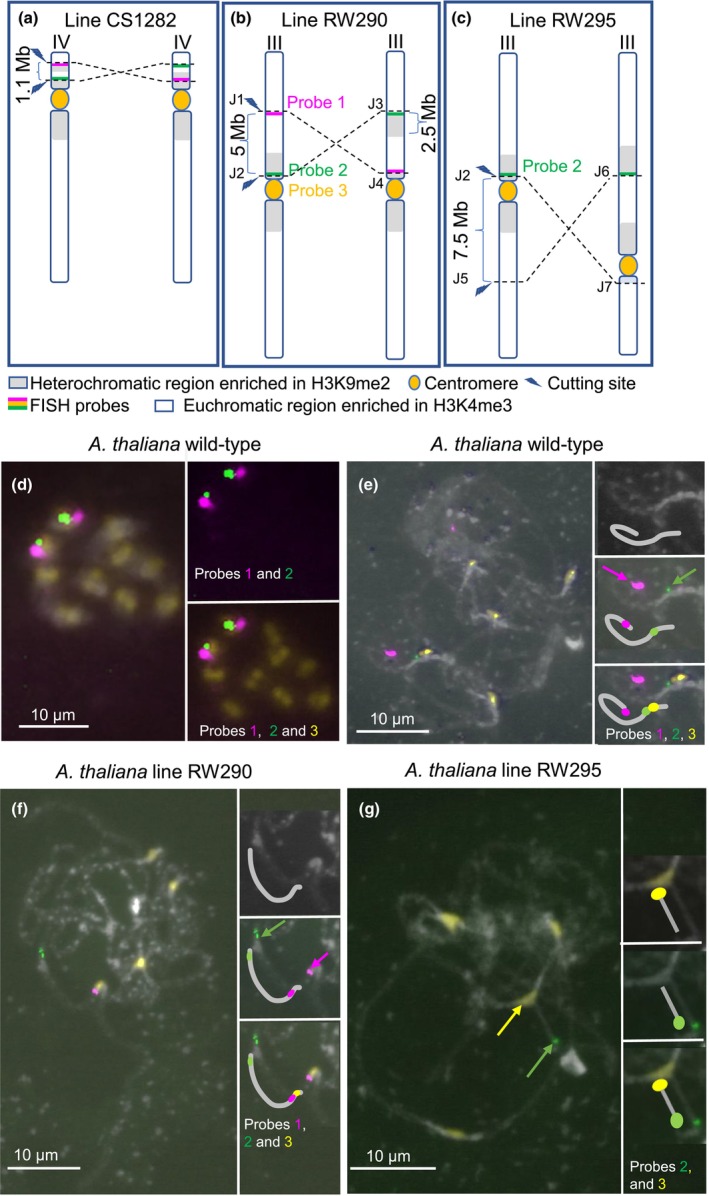
Generated and analyzed CRISPR‐SaCas9‐induced *Arabidopsis thaliana* inversion lines. (a) Line CS1282 with a re‐inversion of the hk4S knob region on chromosome IV (Schmidt *et al*., 2020), (b) line RW290 with a 5‐Mb‐large paracentric and (c) line RW295 with a 7.5‐Mb‐large pericentric inversion of chromosome III. Positions of the inversion breakpoints and applied fluorescent *in situ* hybridization (FISH) probes are indicated. FISH of wild‐type (WT) *A. thaliana* Col‐0 (d) mitotic and (e) pachytene chromosomes with the chromosome III‐specific probes 1 (magenta) and 2 (green) and the centromere‐specific probe 3 (yellow). (f) FISH of line RW290 pachytene chromosomes with the chromosome III‐specific probes 1, 2 and the centromere‐specific probe. Compared with the WT, the green signal moved away from the centromere signal in the inversion line. Instead, the magenta signal moved into the vicinity of the yellow signal. (g) FISH of line RW295 pachytene chromosomes with the chromosome III‐specific probe 1 and the centromere‐specific probe 3. Compared with the WT, the green signal moved away from the centromere signal in the inversion line. Chromatin was counterstained with DAPI. Insets show chromosome III further enlarged. The magenta, green and yellow arrows show the position of probes 1, 2 and 3 signals, respectively.

For comparative RNA‐seq and epigenome analysis, seeds of *A. thaliana* WT (Col‐0), line CS1282 (Schmidt *et al*., 2020) (Fig. 1a) and the newly generated inversion lines were surface‐sterilized and cultured on MS medium. The seedlings were stratified at 4°C for one night and were afterwards cultivated in a growth chamber at 22°C under 16 h : 8 h, light : dark conditions for 2 wk. Two‐week‐old seedlings were harvested and immediately flash‐frozen in liquid nitrogen. Three replicates were collected per line.

#### RNA‐seq analysis

Total RNA was extracted from 100 mg of ground tissue following the protocol of the Quick‐RNA Miniprep kit (Zymo Research, Rezzato, Italy). The integrity of the RNA was assessed using the RNA Integrity Number (RIN). The RNA was sent to BGI (Hong Kong, China) for library preparation and sequencing using the DNBseq PE150 platform. A total of 50 million reads were generated for each sample. The bioinformatics analyses of data were conducted using the Dr Tom network platform provided by BGI (http://report.bgi.com). Quality control measures were applied, and adapter sequences were trimmed using SOAPnuke. Subsequently, the clean data were aligned to the reference genome of *A. thaliana* (Naish *et al*., 2021) using HISAT2 (Kim *et al*., 2015). Differential expression analysis was performed between the inversion lines and the WT using the DESeq2 package (Love *et al*., 2014) with a significance threshold of *Q*‐value ≤ 0.01 and an absolute log_2_ fold change (|log_2_FC|) ≥ 2. Furthermore, the annotated genes were subjected to KEGG pathway enrichment analysis to elucidate their functional significance (Kanehisa *et al*., 2008). Plots of inverted segments and their flanking regions showing the profile of DEGs were generated using pyGenomeTracks (Lopez *et al*., 2021).

#### Chromatin immunoprecipitation followed by sequencing (ChIP‐seq) and analysis

To extract chromatin, 1 g of ground tissues from 2‐wk‐old seedlings was utilized. The ChIP followed the protocol described by Kuo *et al*. (2023), with an increased fixation time of 25 min and the use of a total of 28 cycles for sonication. Antibodies targeting histone H3K4me3 (ab8580; Abcam, Cambridge, UK) and H3K9me2 (ab1220; Abcam) were used to enrich eu‐ and heterochromatin (1 μl of antibody for 100 μl of chromatin), respectively. The concentration of the extracted chromatin was quantified using the QubitTM dsDNA HS Assay kit (Invitrogen). For the preparation of ChIP‐seq libraries, 3 ng of chromatin per sample was used, following the instructions provided by the NEBNext Ultra II DNA Library Prep Kit (NEB, Ipswich, MA, USA; E7645). Subsequently, the libraries were sequenced using DNBseq PE150 by BGI (Hong Kong, China), generating 25 million reads for each library. Three replicates were prepared for each ChIP experiment.

For bioinformatic analysis, the tools available in the Galaxy portal (https://galaxy.ipk‐gatersleben.de) were utilized, as described by Freeberg et al. First, quality control and adapter trimming of ChIP‐seq and input reads were performed with FastQC (v.0.11.8) and Trimmomatic, (v.0.38), respectively. Afterwards, paired‐end reads (2× 150 bp) were aligned to the *A. thaliana* genome (Naish *et al*., 2021) using Bowtie2 with default parameters (Langmead & Salzberg, 2012). MultiBamSummary (v.3.3.0.0.0) was used based on the Pearson's correlation coefficient to assess the similarity between the replicates of each group. Peak calling was executed using Macs2 (v.2.1.1.20160309.6) (Zhang *et al*., 2008) with the following parameters: effective genome size, 119482012; lower mfold, 10; upper mfold, 30; minimum FDR, 0.05; composite broad regions, broad; duplicate tags at the exact same location, 1. Peaks associated with H3K4me3 were analyzed as narrow peaks by adjusting composite broad regions to: no broad. To identify genes marked differentially by H3K4me3 and H3K9me2, the information from two replications was analyzed by Diffbind (Stark & Brown, 2011). Genes with *P*‐value < 0.05 and log_2_ (FC) > 2 were considered differentially methylated regarding H3K4me3 and H3K9me2. The GO and KEGG analysis of the genes associated with identified unique peaks in the inversion lines was conducted using the Database for Annotation, Visualization and Integrated Discovery (David) (da Huang *et al*., 2009; Sherman *et al*., 2022). Normalized coverage BIGWIG files, representing the normalized read coverage across the genome, were generated using BamCompare (v.3.3.0.0.0) by calculating the average log_2_‐ratio of read counts from ChIP over input (Ramírez *et al*., 2016). The generated normalized BIGWIG files were visualized by IGV and pyGenomeTracks to illustrate the distribution of mapped reads across the genome (Robinson *et al*., 2011; Lopez‐Delisle *et al*., 2020). The pathway enrichment bubble plots and KEGG summery plots were generated using the SRplot platform (https://www.bioinformatics.com.cn/en) to illustrate the enrichment of specific biological pathways in the analyzed data (Tang *et al*., 2023).

#### DNA methylation analysis

DNA extraction was performed using the DNeasy® Plant Mini Kit (Qiagen). Three replicates were included for each line. The concentration of DNA was quantified using the Qubit dsDNA broad‐range kit (Invitogen). To assess DNA methylation patterns, the samples were sent to BGI (Hong Kong, China), for WGBS. Before analysis, the raw sequencing data underwent quality assessment using FastQC and subsequent trimming with Trim‐Galore. The reads were aligned to the reference using the Bismark pipeline (Krueger & Andrews, 2011) followed by methylation calling using methylpy with specific parameters: min‐num‐dms 10, min‐cov 5, sig‐cutoff 0.001, dmr‐max‐dist 200. Visualization of the data was facilitated using IGV and pyGenomeTracks (Robinson *et al*., 2011; Lopez‐Delisle *et al*., 2020). Functional annotation of differentially methylated genes was performed using David (da Huang *et al*., 2009; Sherman *et al*., 2022).

## Results

### Targeted engineering of chromosomal inversions by CRISPR/Cas

To determine whether chromosomal rearrangements influence the epigenetic state and transcriptome, we aimed to move a pericentromeric, heterochromatic region of chromosome III into an euchromatic chromosome arm environment. Two kinds of CRISPR/Cas‐engineered chromosomal rearrangements were designed for *A. thaliana* chromosome III: a 5 Mb‐large paracentic (RW290) and a 7.5 Mb‐large pericentric inversion (RW295) (Fig. 1b,c). The cut sites were chosen in a way that they were located close to the boundary between the pericentromere and the 178‐bp satellite array based on sequence information from the SALK 1001 genome browser (http://signal.salk.edu/atg1001/3.0/gebrowser.php). The targeted generation of the inversions followed the protocol by Rönspies *et al*. (2022a). As a first step in generating the respective inversions, suitable spacer sequences, enabling high‐efficiency cutting by SaCas9, had to be identified. This was particularly important when aiming to target the heterochromatic pericentromeric regions of chromosome III, as heterochromatin is less accessible for Cas nucleases (Weiss *et al*., 2022). Several possible target sites were tested by TIDE analysis (Brinkman *et al*., 2014) (Table S1). In the end, three protospacers (PS) were chosen that showed a cutting efficiency of at least *c*. 50%. The PS located close to the centromeric repeats (PS1) was used for the induction of both the para and pericentric inversions. PS1 and PS3 were used for the creation of the paracentric inversion line RW290, and PS1 and PS2 for the creation of the pericentric inversion line RW295. SaCas9, under the control of an egg cell‐specific promoter, was utilized to generate heritable events inducing two simultaneous double‐strand breaks on chromosome III (Steinert *et al*., 2015). To identify plants carrying the rearrangements, 40 T2 plant pools each were analyzed as previously described (Schmidt *et al*., 2020; Rönspies *et al*., 2022a). In the case of the paracentric inversion, four individual plants, each representing independent inversion events, out of 40 T2 pools were identified as positive. The pericentric inversion was found in one plant of 40 T2 pools.

Next, the edited regions of the individual plants were PCR‐amplified and sequenced to analyze the composition of the newly formed inversion junctions. Sequencing data revealed that one of the four plants carrying the paracentric inversion showed a seamless ligation without any sequence loss or gain (Fig. S1A). This plant was chosen for further analysis and propagated as line RW290 in the glasshouse. Analysis of the one plant identified to carry the pericentric inversion revealed an insertion of one nucleotide at the break site (Fig. S1B). To allow further experiments, this plant was propagated as line RW295 in the glasshouse. After seed set, the T3 seeds were harvested and sown on germination medium without antibiotic selection. Afterwards, seedlings were genotyped via PCR using primers specific to the WT and inversion junctions (Table S2). Mendelian segregation of the inversion junctions was confirmed using a chi‐squared test with the critical value χ^2^ (1; 0.95). Plants carrying the inversion in the homozygous state were cultivated in the glasshouse until seed set.

### FISH analysis confirmed the induced chromosomal inversions

To visualize the 5‐Mb‐large paracentric chromosome inversion of line RW290 by FISH, oligo‐painting probes (myTags libraries) were designed based on *c*. 100 kb‐large regions downstream of the cutting site of Cas9 at position 8592 557 (Probe 1) and 100 kb upstream of the bottom cutting site of Cas9 at position 13 496 032 (Probe 2) in the WT (Fig. 1b). By analyzing the order of the probes labeling the adjacent Cas9 cutting sites in combination with a centromere‐specific probe (Probe 3) in the WT and line RW290, the presence of the inversion in line RW290 can be confirmed. To test the specificity of the generated FISH probes, both oligo‐painting probes and the centromere‐specific probe were applied to the mitotic chromosomes of WT *Arabidopsis* Col‐0. Accordingly, both Probes 1 and 2 signals were observed on the same chromosome in the vicinity of the centromere (Fig. 1d). Thus, the colocalization of Probes 1 and 2 on the same chromosome proved the specificity of the generated probes in labeling only chromosome III and not any other chromosomes.

Next, to find out the order and distance of the Probes 1 and 2 signals relative to the centromere, pachytene chromosomes of WT *Arabidopsis* were hybridized with the same combination of FISH probes. In WT, the order of FISH signals generated by Probes 1 and 2 was set up in a way that the magenta signal produced by Probe 1 was distant from the centromere signal while the green signal generated by Probe 2 was in the vicinity of the centromere signal (Fig. 1e). In line RW290, the order of the FISH signals of Probe 1 and 2 was, due to the chromosome III‐specific inversion, changed in a way that the green signal moved away from the centromere signal and the magenta signal moved closer to the centromere signal (Fig. 1f). Thus, the FISH analysis confirmed that line RW290 carries a CRISPR/Cas9‐induced inversion on chromosome III.

To prove the presence of the 7.5‐Mb‐large pericentric inversion, FISH was performed with Probe 1 and the centromere probe on pachytene chromosomes of line RW295 and, for comparison, WT. FISH analysis of the WT chromosomes revealed Probe 1 signals near the centromere signal (Fig. 1e). On the other hand, FISH of line RW295 with the same probes showed that the green signal from Probe 1 had moved away from the centromere of chromosome III (Fig. 1g). Therefore, the FISH results proved that line RW295 indeed carried the inversion in this region.

### After induction of chromosomal inversions, the global distribution of histone marks specific to eu‐ and heterochromatin remains unaltered

To investigate the impact of the generated chromosome segment inversions in the earliest homozygous generation (generation T5) on the epigenetic status of the chromosomes, the distribution of post‐translational histone marks typical for eu‐ (H3K4me3) and heterochromatin (H3K9me2) between WT *A. thaliana* and the inversion lines was compared. The inversion in line RW290 resulted in the displacement of a 2.5‐Mb‐long heterochromatic region from the pericentromeric region into the euchromatic long arm of chromosome III. In line RW295, a 7.5‐Mb‐long region, including the centromere, was inverted, changing the submetacentric chromosome III into an acrocentric chromosome. Consequently, the repositioning of eu‐ and heterochromatic regions in the inversion lines caused the formation of new eu‐/heterochromatic boundaries. Besides RW290 and RW295, line CS1282 (Schmidt *et al*., 2020) was included in this study as a control, featuring a 1.1‐Mb‐long inversion that moved a heterochromatic region into the heterochromatic pericentromeric region of chromosome IV (Fig. 1a).

**Fig. 2:**
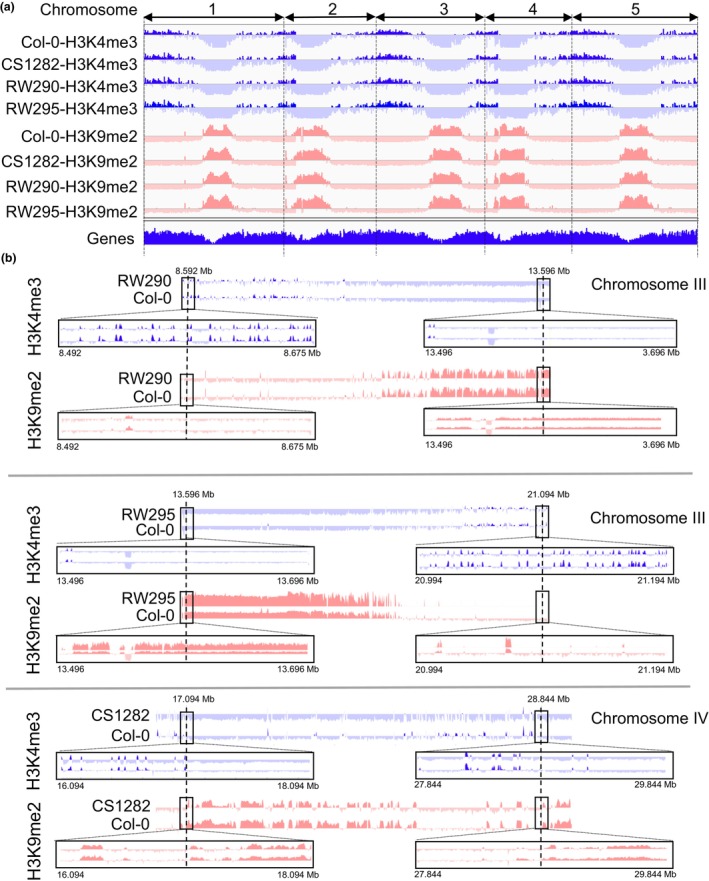
Global distribution of histone marks specific to eu‐ and heterochromatin remains unaltered following the induction of chromosomal inversions. (a) Similar genome‐wide distribution of eu‐ (H3K4me3) and heterochromatic (H3K9me2) histone marks between lines RW290, RW295, CS1282 and wild‐type (WT) *Arabidopsis thaliana* Col‐0. (b) Further resolved distribution of H3K4me3 and H3K9me2 marks within the inversion segments and proximal to the breakpoints (±100 kb). To allow visual comparison of epimarks along the chromosomes, the inverted chromosome segments of all three lines are shown in an inverted orientation. The comparisons are not in scale. Note, Supporting Information Fig. S3 shows the plotted data against a physically rearranged genome assembly.

ChIP‐seq with H3K4me3‐ and H3K9me2‐specific antibodies was performed using 2‐wk‐old seedlings of all lines to investigate the epigenetic consequences of the chromosome segment inversion. Three replicates were prepared for each genotype and antibody. The sample correlation test demonstrated a high similarity between the ChIP and input replicates. For line CS1282, only two replicates were deemed valid (Fig. S2). To allow visual comparison of epimarks along the chromosomes, the inverted chromosome segments of all three lines are shown in an inverted orientation (Fig. 2) and also shown on the *in silico*‐inverted reference genome for lines RW290 and RW295 (Fig. S3). In other words, the chromosome segment inversions are masked. Comparison of the ChIP‐seq data between inversion lines and WT revealed that none of the inversions affected the global distribution of H3K4me3 and H3K9me2 epimarks (Fig. 2a). In all lines, the chromosome arms were enriched in H3K4me3. At the same time, the pericentromeric regions showed a H3K9me2 enrichment. Also, at higher resolution, a comparable distribution was observed for both epimarks between the inverted chromosome segments and WT in the proximal regions (±100 kb) to the breakpoints of all three inversions (Fig. 2b).

Although none of the chromosomal inversions changed the global distribution of epimarks, 29, 25 and 45 genes of lines RW290, RW295 and CS1282 changed in their histone H3K9me2 patterns compared with WT, respectively (Fig. S4A–C). Only genes that were affected in all three replicates were considered. A slightly higher number of the altered genes was found in the case of H3K4me3. 31, 44 and 76 genes of lines RW290, RW295 and CS1282 changed in their histone H3K4me3 patterns compared with the WT, respectively. Further, all affected genes, reflecting a small fraction of the total number of genes, were distributed over the entire genome and not restricted to the inverted chromosome segments. Thus, except for minor exceptions, the global distribution of histone marks specific to eu‐ and heterochromatin remained unaltered following the induction of chromosomal inversions.

### The global DNA methylome remains preserved after the induction of chromosomal inversions

**Fig. 3:**
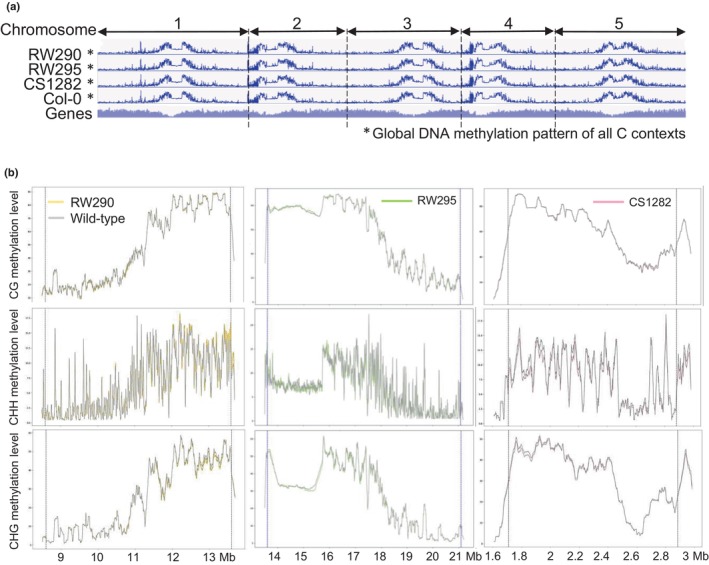
Global DNA methylome remains preserved following the induction of the chromosome segment inversions. (a) Global DNA methylation pattern of all C contexts over all chromosomes of the *Arabidopsis thaliana* lines carrying an inversion compared with the wild‐type (WT). (b) Comparison of different C context methylation levels compared with WT Col‐0 in the area of the inversion and the ±100 kb flanking regions. The dotted blue line indicates the breakpoint positions. To allow visual comparison of DNA methylation marks along the chromosomes, the inverted chromosome segments of all three lines are shown in an inverted orientation.

To investigate the effect of the chromosome segment inversions on the DNA methylome, WGBS was performed. To allow a visual comparison of methylated DNA, again, the inverted chromosome segments of all three lines are shown in an inverted orientation (Fig. 3a). The global DNA methylation profile of all three lines compared with WT plants was the same. Similarly, a detailed comparison of methylated CG, CHG and CHH sites in the inverted chromosome and flanking regions (±100 kb) showed no alterations in all three lines (Fig. 3b). However, hundreds of differentially methylated regions (DMRs) were found for each C context, which were distributed across the entire genome. In total, 986, 729 and 901 DMRs were explicitly identified for RW290, RW295 and CS1282, respectively (Fig. S5A,B). The KEGG pathway summary of identified DMRs showed that the identified DMRs are mostly involved in metabolic pathways responsible for providing energy or involved in defense (Fig. S6A–C). Thus, except for minor exceptions, the global DNA methylome remained preserved following chromosomal restructuring.

### Gene expression does not change after induction of chromosome segment inversions

**Fig. 4:**
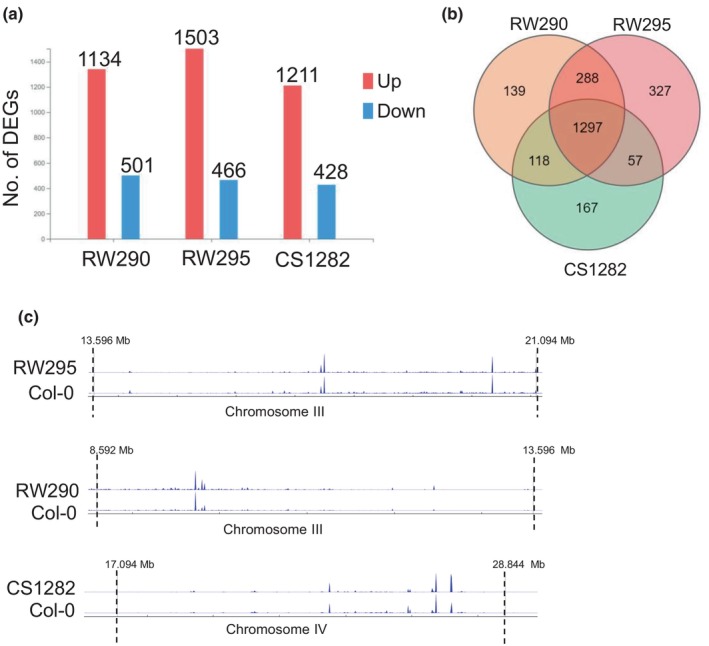
Gene expression changed to some extent after the induction of chromosome segment inversions in *Arabidopsis thaliana*. (a) More than 1000 differentially expressed genes (DEGs) were identified in each inversion line. (b) From the total number of DEGs, a modest number of DEGs, specifically 139, 167 and 327 genes were recognized to be specific to lines RW290, CS1282 and RW295, respectively. A total of 1297 DEGs were shared in all inversion lines. (c) Gene expression profile of RW290, CS1282 and RW295 in the inverted and flanking regions to the break points. The expression profile of each line compared to the control was not affected by the inversion events in the inversion segments and the ±100 kb flanking regions. To allow visual comparison of DEGs along the chromosomes, the inverted chromosome segments of all three lines are shown in an inverted orientation.

**Fig. 5:**
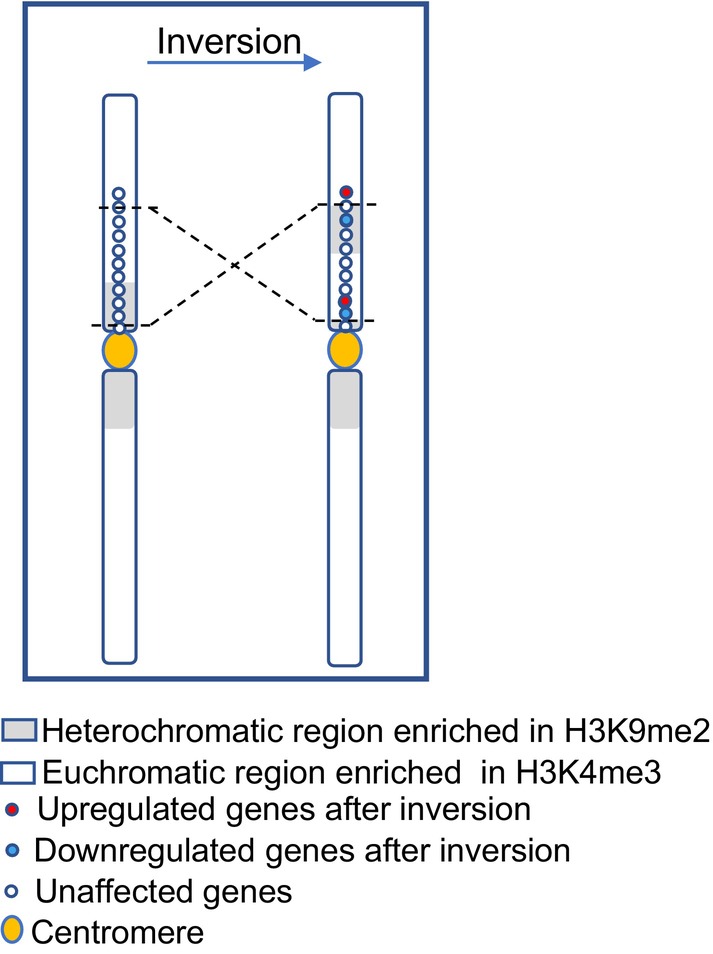
Model of the effect of a chromosome segment inversion on gene expression. Despite the formation of new eu/heterochromatic borders in the inversion lines, the genes located near the inversion borders mostly did not alter their expression due to the juxtaposition to eu‐ or heterochromatin except for a few genes. The activity and position of the genes are shown as red (downregulated), blue (upregulated) and white (not affected) points.

Finally, it was determined by comparative RNA‐seq whether chromosome segment inversions alter gene expression dynamics. The PCA demonstrated a strong correlation between the replicates of each line and the distinct differences between the inversion lines and the WT (Fig. S7). In each line, over 1500 differentially expressed genes (DEGs) were detected, representing 5.9–7.1% of the total transcriptome (Fig. 4a). Only a small number of these genes was specific to each line (Fig. 4b). In total, 0.5%, 0.6% and 1.18% of DEGs were observed only in lines RW290, CS1282 or RW295, respectively. Therefore, the three lines shared the majority of DEGs. The KEGG pathway enrichment analysis of the DEGs revealed their involvement in metabolism or defense pathways (Fig. S8A–C). A detailed gene activity comparison between the inverted chromosome regions and flanking regions (±100 kb) and WT showed that based on the expression profile of each line, none of them were affected by the inversion events (Fig. 4c). In total, 4, 38 and 1 DEGs were identified within the inverted segments in lines RW290, RW295 and CS1282, respectively. Again, most of the identified DEGs within the inverted region were involved in the regulation of metabolic pathways or defense mechanisms. Unexpectedly, the expression profile of the identified genes was not influenced by the juxtaposition of the new euchromatic/heterochromatic borders (Table S3). In conclusion, except for minor exceptions, the global transcriptome and epigenome remained preserved following chromosomal restructuring, at least in the following generations (Fig. 5).

## Discussion

Previous studies analyzing naturally occurring inversions revealed that this type of chromosomal structural variation can affect the gene expression of adaptive and agronomic traits due to modifying large regulatory domains (Naseeb *et al*., 2016; Hu *et al*., 2024) as well as alter genetic or epigenetic environments near the breakpoints (Wesley & Eanes, 1994). In addition, in numerous species, inversions play a role in driving genome evolution (Wellenreuther & Bernatchez, 2018). Indeed, different kinds of chromosome segment inversions have been found in many cultivars of prominent crop species such as rice (Zhou *et al*., 2023), maize (Crow *et al*., 2020), barley (Jayakodi *et al*., 2020) and other species (Chen *et al*., 2024). So far, only historic chromosomal rearrangements that had occurred naturally could be investigated in this regard in plants. Now that the CRISPR/Cas‐based chromosome engineering technique was recently established, predefined chromosome rearrangements can be induced, and their genetic and epigenetic consequences can now be analyzed directly after their occurrence (Rönspies *et al*., 2021). This technology is especially attractive for plant breeding, as the induction of targeted chromosomal rearrangements can be useful for manipulating genetic linkages (Puchta & Houben, 2024). By inducing reciprocal translocations between chromosomes, genetic linkages can either be broken or established (Beying *et al*., 2020). On the other hand, chromosomal rearrangements also play a role in the modulation of meiotic recombination as they suppress crossovers in the rearranged area during meiosis. Therefore, they often present a hurdle for plant breeders since they rely on natural meiotic recombination to generate new favorable allelic combinations (Termolino *et al*., 2019). Thus, the possibility to reverse, for example, inversions to make recombination‐dead regions of the chromosome accessible for genetic exchange again is of great value for plant breeding (Schwartz *et al*., 2020). Indeed, chromosome engineering could be used to revert a naturally derived 1.17 Mb inversion, called hk4*S*, on chromosome 4 in *A. thaliana*. By recombination analysis, it could be shown that recombination in this previously recombination‐cold area can be restored (Schmidt *et al*., 2020). In a second study, to determine whether targeted suppression of recombination can be achieved in a large part of the genome, almost an entire chromosome was inverted in *A. thaliana* (Rönspies *et al*., 2022b). The subsequent recombination analysis showed that, indeed, crossovers can be supressed in almost an entire chromosome by chromosome engineering (Rönspies *et al*., 2022b).

On the other hand, the application of chromosome engineering makes it possible to answer long‐standing basic research questions, such as defining the role of the chromosomal position of a DNA sequence on its epigenetic stability and gene activity. To address this question, in this study, two differently sized inversions were induced that purposely moved heterochromatic, pericentric sequences into an euchromatic chromosome arm context. This made it possible to test for the first time whether or not the epigenetic landscape, as well as gene expression levels, remains preserved following chromosomal restructuring, at least in the following generations.

The consequences of PEV, as observed in *Drosophila*, or TPE, as detected in budding yeast (Bao *et al*., 2007; Kitada *et al*., 2012), arising from the occurrence of chromosomal rearrangements, are prominent examples of the chromosome position effect on the regulation of gene expression. The underlying molecular mechanisms for the impact of the chromosome position on gene expression have been attributed to several factors, including changes in the epigenetic environment of the rearranged region (Bao *et al*., 2007; Fournier *et al*., 2010; Kitada *et al*., 2012). In the case of PEV, heterochromatin formation depends on multiple interactions between H3K9 methyl*trans*ferases (HKMTs), heterochromatin protein 1 (HP1a) and methylation of histone H3 at lysine 9 (H3K9me2/3) (Elgin & Reuter, 2013). The heterochromatin formation in PEV can range from 10 kb to hundreds of kb in *Drosophila*, depending on the specific position (Haynes *et al*., 2007). In plants, the only case of PEV has been reported in *Oenothera blandina* (Catcheside, 1939). However, the underlying mechanism of this phenomenon is not well‐described. By contrast, our data show that heterochromatinization of inverted euchromatic segments juxtaposed to heterochromatic regions does not occur, even within up to 100 kb around the chromosome segment breakpoints, in the *Arabidopsis* plants that were generated in this study. The newly established eu‐/heterochromatic borders at the inversion points retained their WT epigenetic marks, including histone and methylation marks, in all three analyzed inversion lines. Our finding is consistent with the effects of the chromosome segment inversion observed in the hk4S genotype of *A. thaliana* and a synthetic chromosome in moss (semi‐syn18L) (Fransz *et al*., 2016; Chen *et al*., 2024), indicating that the epigenetic marks are not defined by the chromosomal position in the genomic regions chosen for our experiment in *Arabidopsis*. Whether the epigenome is faithfully restored after DNA damage repair is still a matter of debate (Dabin *et al*., 2016, 2023). Our analysis revealed that the epigenome of the CRISPR/Cas9 cutting sites did not change after repair, which is in accordance with research that investigated the methylation pattern of several target and off‐target genes in *Arabidopsis* edited by Cas9 (Lee *et al*., 2019). Changes in the DNA methylation profile after the occurrence of chromosomal rearrangements were reported in the case of naturally inverted segments in human cells (Jamil *et al*., 2019; Carreras‐Gallo *et al*., 2022) and *Brassica napus* hybrids (Boideau *et al*., 2022). Obviously, the regions selected for the inversion induction in our experiment are not inversion‐prone positions in contrast to the ones described in human cells (Jamil *et al*., 2019; Carreras‐Gallo *et al*., 2022; Hazarika *et al*., 2022). In human cells, inversions can cause diseases, although these inversions do not alter the coding sequence. Some inversions are reported to influence the methylation profile of the inverted segment and its borders (Jamil *et al*., 2019; Carreras‐Gallo *et al*., 2022). Therefore, by changing DNA methylation, the activity of genes was affected as well. (Carreras‐Gallo *et al*., 2022). In plants, the effect of chromosomal rearrangements has so far only been studied in the case of events that occurred many generations earlier so that inversion‐independent subsequent events could be responsible for the changes, such as described in *B. napus* (Jamil *et al*., 2019). Our findings, however, indicate that in the first few generations following the introduction of the inversions (T5), the chromatin context was not affected. In light of this result, it would also be interesting to analyze later generations in the future.

The perturbation of the interaction of *cis*‐ and transregulatory elements or the variation of genetic regions close to the inversion breakpoints are other reasons for possible changes in the gene activity due to the reordering of the genes' positions in the genome (Naseeb *et al*., 2016; Lavington & Kern, 2017; Crow *et al*., 2020). In our study, the gene expression profiles showed only slight changes following the chromosomal restructuring, such as those observed in the case of the hk4S inversion in *A. thaliana* (Fransz *et al*., 2016). In the case of the hk4S event, the inversion was induced naturally by Vandal5 transposon elements, which generated a clean split in the genes near the breakpoint (Fransz *et al*., 2016). In this study, the cutting sites of the CRISPR/Cas system lie far beyond the regulatory regions of genes (at least for RW290; Fig. S9). Additionally, genotyping confirmed that there is no genetic variation between the inversion and WT plants. This finding provides a reliable condition for focusing only on the effect of the chromosomal position on the regulation of genes. Therefore, the observed 100–300 DEGs in our lines did likely not arise due to the disruption of genes or their regulatory elements near the breakpoints, as they were distributed throughout the genome rather than restricted to the inversion segments or their surroundings. On the other hand, it is possible that the chromosomal rearrangements affected the 3D organization of the chromatin and that, subsequently, the expression of the underlying genes was slightly affected. The high number of common DEGs between the three lines could be due to the regulation of overlapping transcriptional networks or pathways controlled by the DEGs unique to each line. Our observation aligns with the outcome of a study that used a FRT‐based recombination system to induce defined 160–265 kb‐long chromosomal inversions in *Drosophila* (Meadows *et al*., 2010). Comparative analysis of inverted vs WT genotypes revealed no significant differences in the expression of neighboring genes. A similar observation was obtained after analysis of *Drosophila* lines possessing highly rearranged chromosomes. Despite major changes in genome organization, only a few hundred genes showed moderate expression changes (Ghavi‐Helm *et al*., 2019). In mice, it has been demonstrated that induced chromosome fusions affect the radial distribution of chromosome territories. However, these perturbations only led to slight changes in gene expression (0.33%), with DEGs distributed globally across the genome rather than being confined to the fused chromosomes (Wang *et al*., 2023). The fact that the epigenetic status of the inverted sequences was not remodeled in the generations following the occurrence of the inversion events gives us the opportunity to address another important unsolved question in the future: Is the efficiency of meiotic recombination mainly determined by the position or the heterochromatic state of the respective region of the chromosome? Heterochromatic regions close to the centromere are depleted of crossovers compared with the euchromatic chromosome arms (Naish *et al*., 2021). Establishing similar inversions in another *A. thaliana* cultivar besides Col‐0 could help determine, through crossing and SNP analysis, whether large heterochromatic regions suppress crossovers equally when moved within the chromosome compared with their original pericentric positions. This will make it possible to define whether the chromosomal position influences crossover frequencies.

Finally, the fact that targeted inversions – at least in the tested cases – change neither the epigenetic state nor the transcriptome in plants is encouraging news for future applications of chromosome engineering in crop breeding. For trait improvement, large‐scale inversions have already been induced using Cas9 in corn (Schwartz *et al*., 2020) and rice (Lu *et al*., 2021). Thus, no unwanted epigenetic side effects can endanger the envisaged breeding success or raise consumer concerns. Indeed, the EU Commission suggested to exclude nature‐identical inversions from future GMO regulation in Europe (Puchta, 2024).

## Competing interests

None declared.

## Author contributions

AH, SK, R Hinrichs, MR and HP designed research; R Hinrichs generated CRISPR/Cas‐engineered chromosome segment inversions; SK performed FISH, ChIP‐seq and performed bioinformatic analysis; R Haghi performed bioinformatic analysis; and all authors wrote the paper.

## Disclaimer

The New Phytologist Foundation remains neutral with regard to jurisdictional claims in maps and in any institutional affiliations.

## Supporting information


**Fig. S1** Molecular nature of the *Arabidopsis thaliana* wild‐type and inversion junctions of the inversion lines.
**Fig. S2** Sample correlation test between replicates of chromatin immunoprecipitation and input samples.
**Fig. S3** Global distribution of histone marks specific to eu‐ and heterochromatin mapped to *in silico* inverted reference genome of *Arabidopsis thaliana*.
**Fig. S4** Number of genes with differentially K4‐ and K9‐methylated histone marks demonstrated for three replicates of ChIP‐seq in line RW290, CS1282 compared with wild‐type.
**Fig. S5** RW295, RW290 and CS1282 inversion line‐specific genome‐wide distributed differentially methylated regions.
**Fig. S6** KEGG pathway summary of identified differentially methylated regions in line RW290 and Rw295 and CS1282.
**Fig. S7** PCA test comparing the transcriptome of *Arabidopsis* lines RW295 and RW290 with the wild‐type.
**Fig. S8** KEGG pathway enrichment histogram of recognized differentially expressed genes for lines RW290, RW295 and CS1282.
**Fig. S9** Distance and orientation of nearby genes to the breakpoints of the CRISPR/Cas cutting sites J1, J2 and J5.
**Table S1** List of the protospacers tested for the establishment of both inversions.
**Table S2** Sequences that were used as protospacers, TIDE primers and PCR primers for amplifying the inversion and wild‐type junctions.
**Table S3** Identified differentially expressed genes within the inverted regions of RW290, RW295 and CS1282.
Please note: Wiley is not responsible for the content or functionality of any Supporting Information supplied by the authors. Any queries (other than missing material) should be directed to the *New Phytologist* Central Office.

## Data Availability

The raw read data for this study have been deposited in the European Nucleotide Archive (ENA) at EMBL‐EBI under accession no. PRJEB81173 (https://www.ebi.ac.uk/ena/browser/view/PRJEB81173).
