# Supplementary material for: Epigenetic state and gene expression remain stable after CRISPR/Cas‐mediated chromosomal inversions
Source: New Phytol. 2025 Jan 29;245(6):2527–39. doi: 10.1111/nph.20403 (PMC11840415; doi:10.1111/nph.20403)
Supplement: Supplementary file 1 — Fig. S1 Molecular nature of the Arabidopsis thaliana wild‐type and inversion junctions of the inversion lines. Fig. S2 Sample correlation test between replicates of chromatin immunoprecipitation and input samples. Fig. S3 Global distribution of histone marks specific to eu‐ and heterochromatin mapped to in silico inverted reference genome of Arabidopsis thaliana. Fig. S4 Number of genes with differentially K4‐ and K9‐methylated histone marks demonstrated for three replicates of ChIP‐seq in line RW290, CS1282 compared with wild‐type. Fig. S5 RW295, RW290 and CS1282 inversion line‐specific genome‐wide distributed differentially methylated regions. Fig. S6 KEGG pathway summary of identified differentially methylated regions in line RW290 and Rw295 and CS1282. Fig. S7 PCA test comparing the transcriptome of Arabidopsis lines RW295 and RW290 with the wild‐type. Fig. S8 KEGG pathway enrichment histogram of recognized differentially expressed genes for lines RW290, RW295 and CS1282. Fig. S9 Distance and orientation of nearby genes to the breakpoints of the CRISPR/Cas cutting sites J1, J2 and J5. Table S1 List of the protospacers tested for the establishment of both inversions. Table S2 Sequences that were used as protospacers, TIDE primers and PCR primers for amplifying the inversion and wild‐type junctions. Table S3 Identified differentially expressed genes within the inverted regions of RW290, RW295 and CS1282. Please note: Wiley is not responsible for the content or functionality of any Supporting Information supplied by the authors. Any queries (other than missing material) should be directed to the New Phytologist Central Office. [file NPH-245-2527-s001.pdf]

Article title: Epigenetic state and gene expression remain stable after CRISPR/Cas-mediated chromosomal inversions

Authors: Solmaz Khosravi, Rebecca Hinrichs, Michelle Rönspies, Reza Haghi, Holger Puchta and Andreas Houben
Article acceptance date: 17 December 2024

A

Wild-type J1 ATAAACGATACCCAACTGATAAAACATCCATGAAGATGTTTATT

Wild-type J2 GCTTTCTTATCTTAGTATAAATAAGTCTGTAAGGGTTTCGCCCT

RW290 J3 ATAAACGATACCCAACTGGACTTATTTATACTAAGATAAGAAAG

RW290 J4 AATAAACATCTTCATGGATGTTTTATTGTAAGGGTTTCGCCCTA

B

Wild-type J2 GCTTTCTTATCTTAGTATAAATAAGTCTGTAAGGGTTTCGCCC

Wild-type J5 TTAGCTCTTGACCCTCGCTCAATCTCACGGCCATGCCGTCCTA

RW295 J6 GCTTTCTTATCTTAGTATAAATAAGTCTAGCGAGGGTCAAGAG

RW295 J7 TTAGGGCGAAACCCTTACAACAATCTCACGGCCATGCCGTCCT

Figure S1. Molecular nature of the wild-type and inversion junctions (J) of the inversion lines. A) Molecular nature of the wild-type (wild-type J1 and J2) and inversion junctions (RW290 J3 and J4) for production of RW290. The first two lines show the natural wild-type conformation and the last two lines show the nucleotide composition of the inversion junctions in line RW290 as determined by Sanger sequencing. The protospacer adjacent motif (PAM) of the 5´-sequence is highlighted in pink and the corresponding spacer sequence is highlighted in blue. The PAM of the 3´-sequence is highlighted in gray and the corresponding spacer sequence is highlighted in green. B) Molecular nature of the wild-type (wild-type J2 and J5) and inversion junctions (RW295 J6 and J7) for production of RW295. The first two lines show the natural wild-type conformation and the last two lines show the nucleotide composition of the inversion junctions in line RW295 as determined by Sanger sequencing. The PAM of the 5´-sequence is highlighted in gray and the corresponding spacer sequence is green in blue. The PAM of the 3´-sequence is highlighted in pink and the corresponding spacer sequence is highlighted in yellow. The blue letters represent the insertion of one nucleotide at the break site. Since PS1 was used to generate both inversions, the wild-type J2 sequence is identical in A) and B).

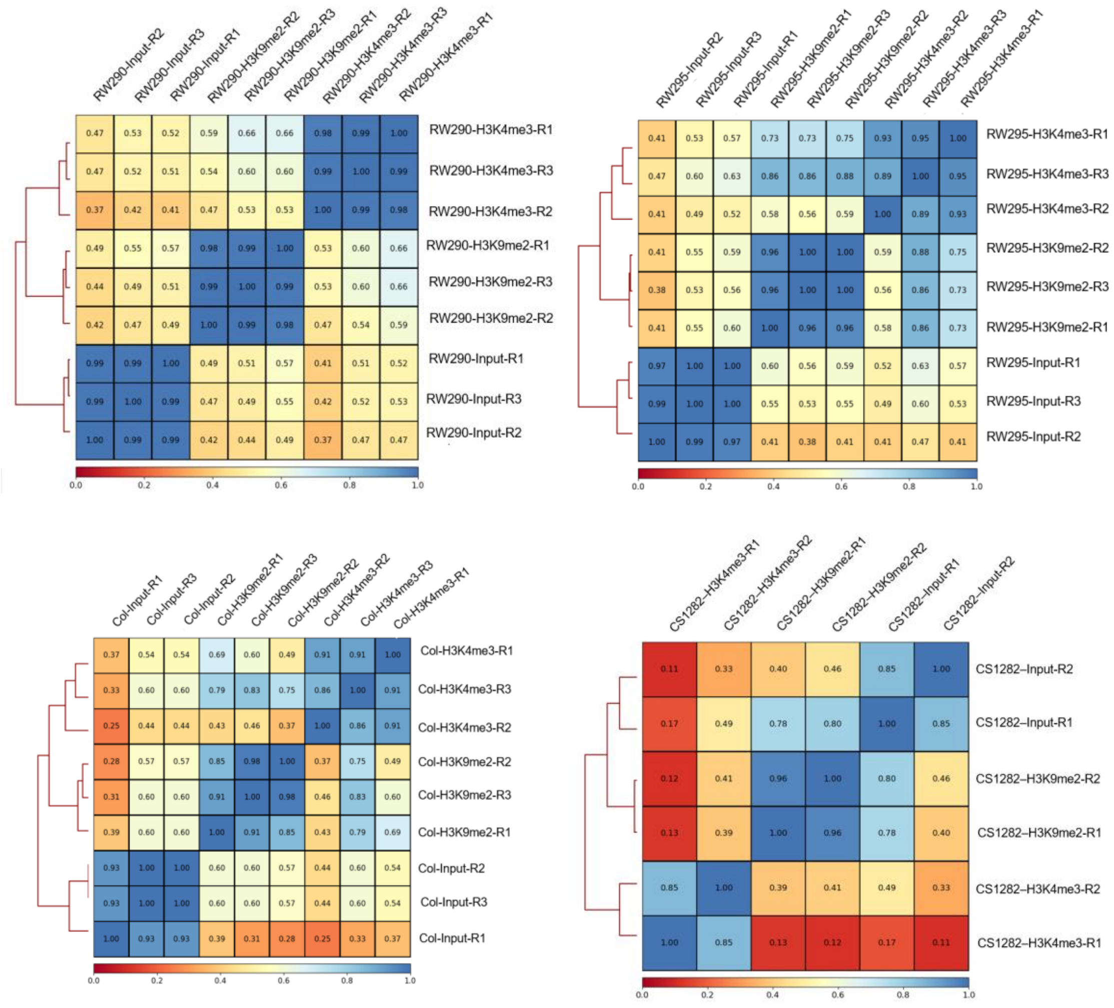

Figure S2. Sample correlation test between replicates of ChIP and Input samples.

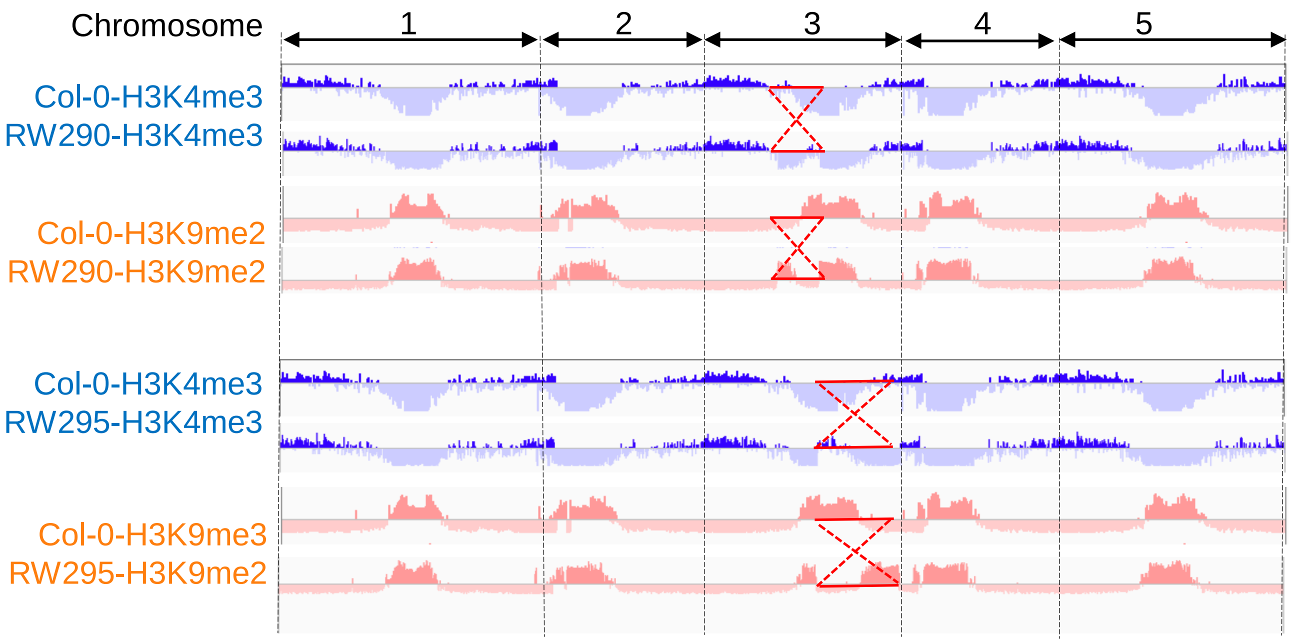

Figure S3. Global distribution of histone marks specific to eu- and heterochromatin mapped to *in silico* inverted reference genome. Red lines show the location of inversions.

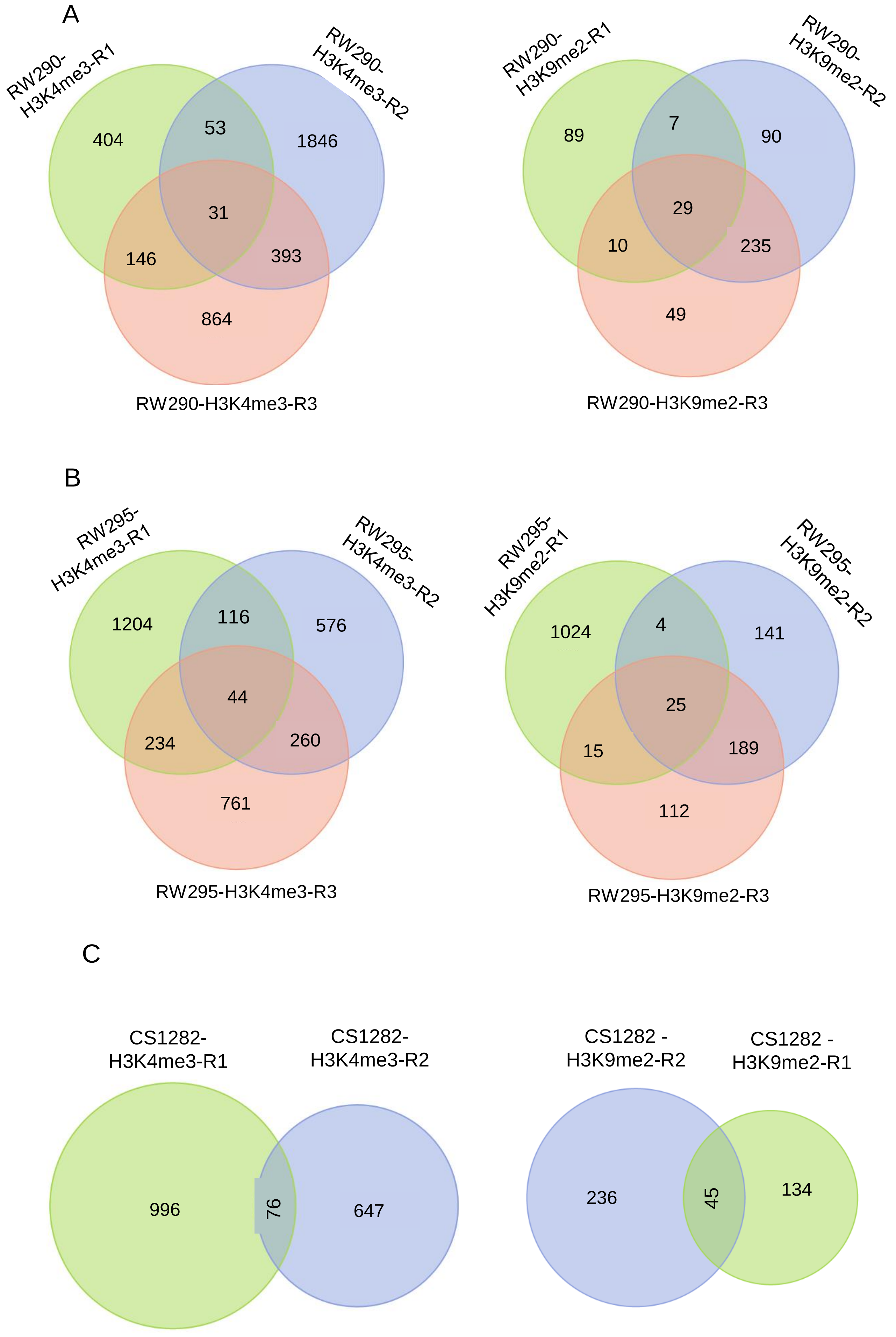

Figure S4. Number of genes with differentially K4 and K9 methylated histone marks demonstrated for three replicates of ChIP-seq in line A) RW290, B) CS1282 compared to wild-type.

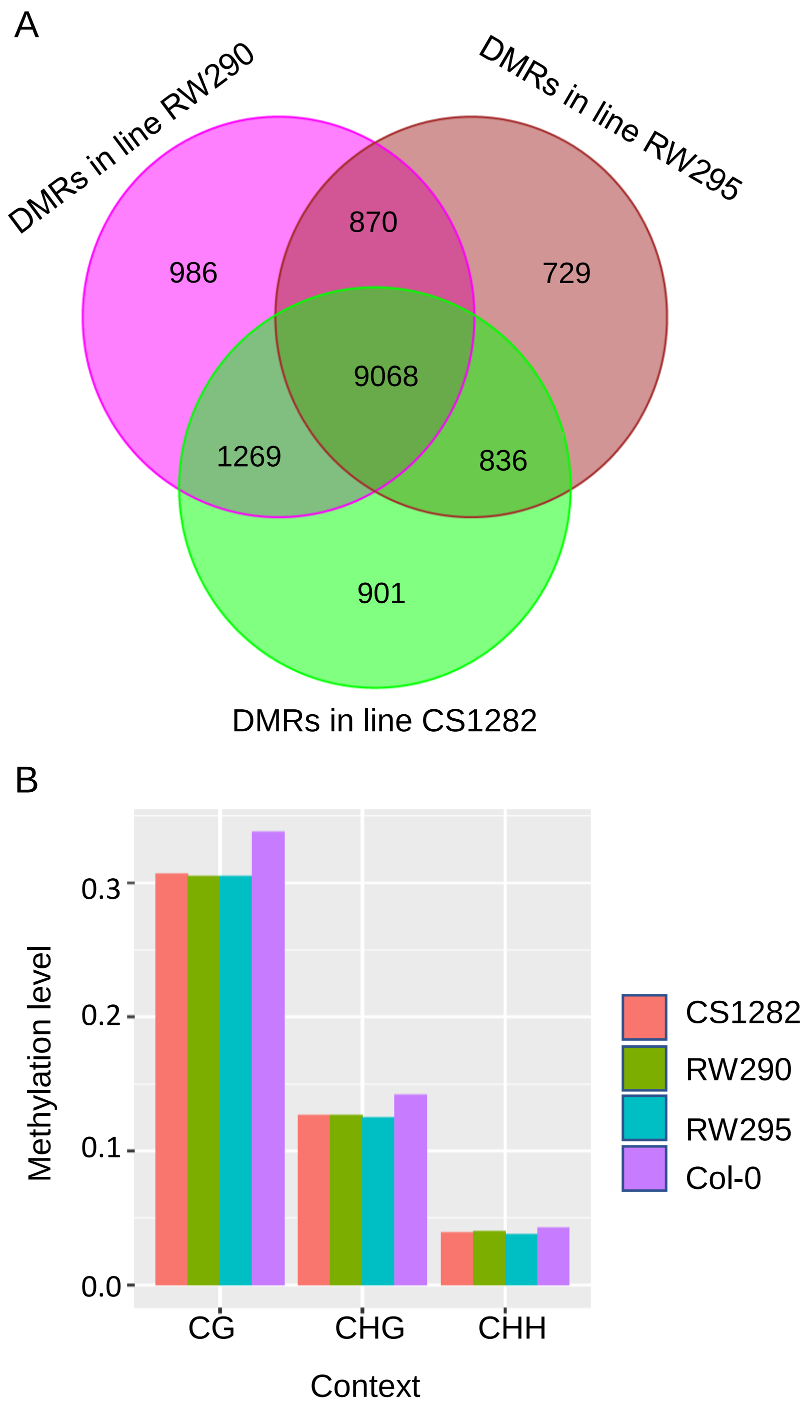

Figure S5. RW295, RW290 and CS1282 inversion line-specific genome-wide distributed DMRs. A) Comparison of the number of shared and inversion line-specific DMRs identified in each line. B) Comparison of methylation level in different C contexts between wild-type and inversion lines. Compared to wild-type, the inversion lines show a slight reduction in methylation levels of CG, CHG and CHH.

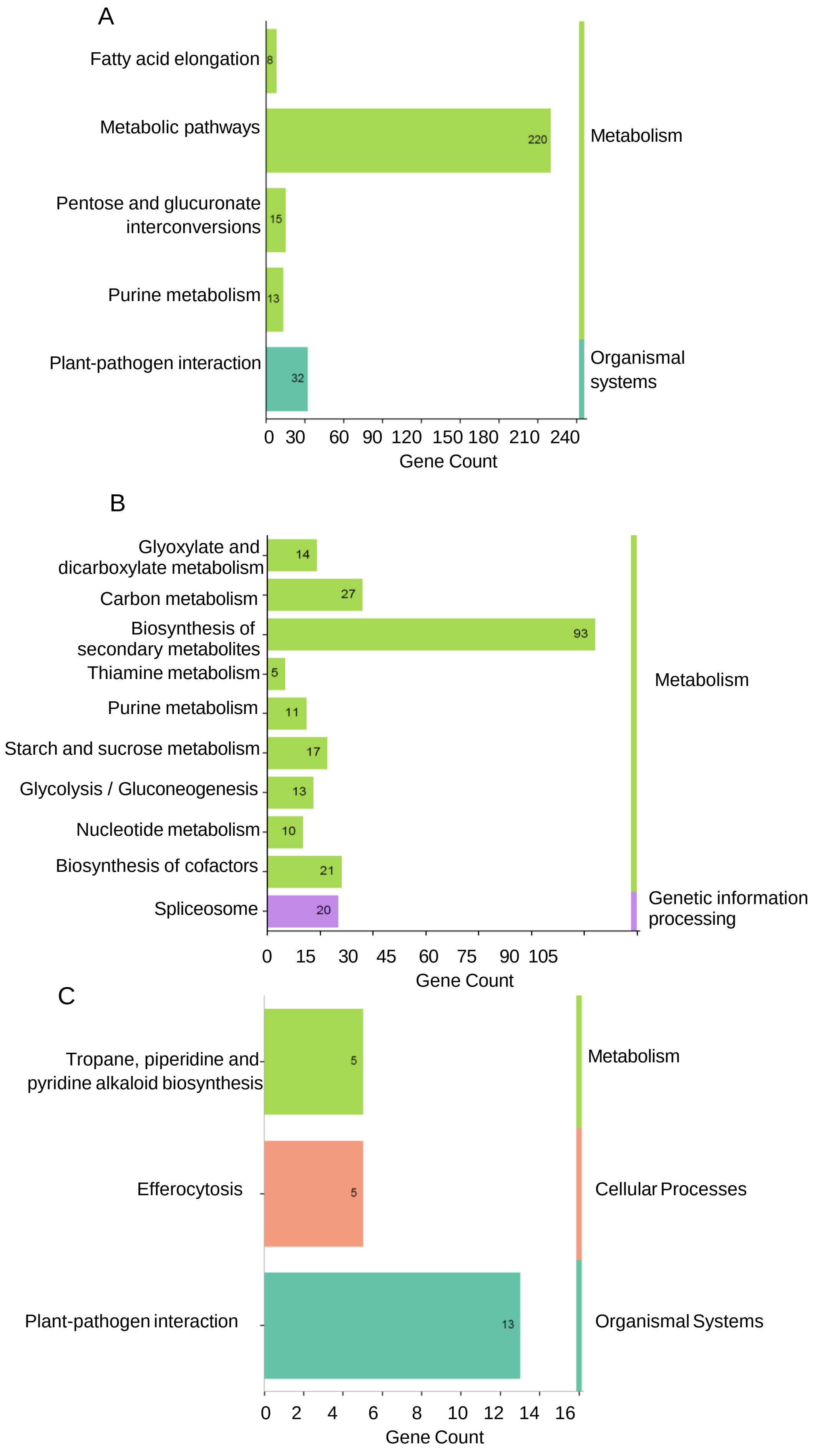

Figure S6. KEGG pathway summary of identified DMRs in A) line RW290 and B) Rw295 and C) CS1282. The identified genes are mostly involved in the regulation of metabolism pathways.

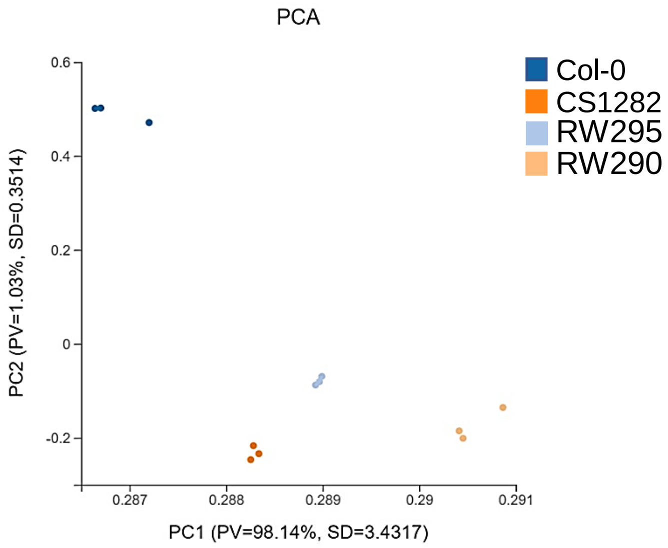

Figure S7. PCA test comparing the transcriptome of *Arabidopsis* lines RW295 and RW290 with the wild-type.

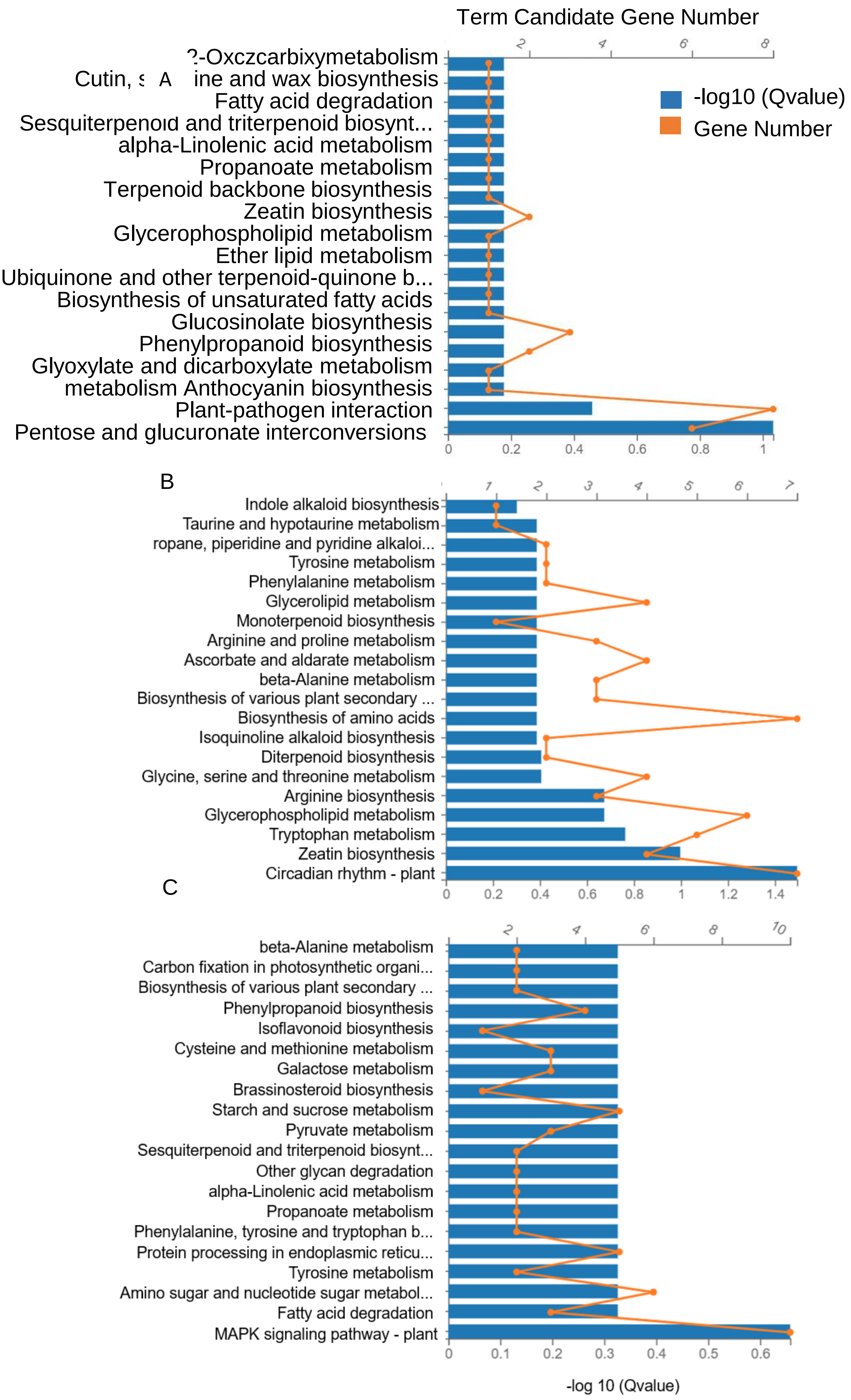

Figure S8. KEEG pathway enrichment histogram of recognized DEGs for lines A) RW290, B) RW295 and C) CS1282.

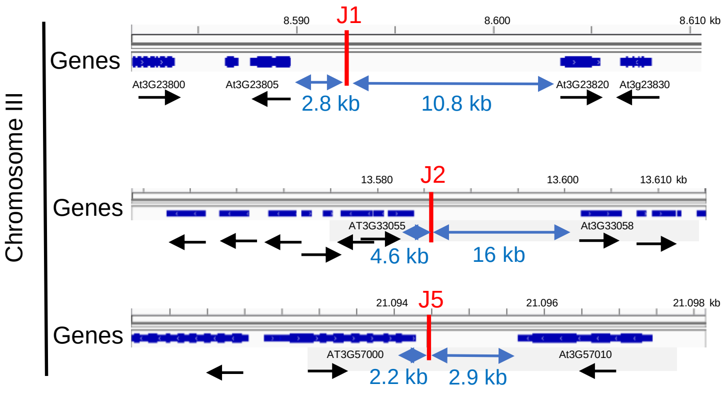

Figure S9. The distance and orientation of nearby genes to the breakpoints of the CRISPR/Cas cutting sites J1, J2 and J5.

Table S1. List of the protospacers tested for the establishment of both inversions. Shown here are the names and positions of the protospacers on chromosome 3 (TAIR10 as reference genome), the cutting efficiency and the sequence of the protospacers. Among the tested protospacers, the ones with highest cutting efficiency were used to generate the inversions. The protospacer RW67/68 (PS1) was used in combination with RW108/109 (PS2) to generate line RW295 and in combination with RW185/186 (PS3) to establish line RW290.

Name (Protospacer)	Position (TAIR10)	Efficiency	Sequence
RW65/66	13,584,987	27.8%	TCTGGGCTAGATATGACTAG
RW67/68 (PS1)	13,585,829	69.10%	CTTAGTATAAATAAGTCTGT
RW69/70	13,584,800	11.4%	AAGCAGCTACTGGGGTTAGC
RW108/109 (PS2)	21,094,474	87.2%	GCATGGCCGTGAGATTGAGC
RW110/111	21,094,833	48.7%	TGAAATGAACCAAACAAAAC
RW185/186 (PS3)	8,592,547	44%	CTTCATGGATGTTTTATCAG
RW187/188	8,592,854	8.5%	CATGATTCAAGAAAATATAT
RW189/190	8,592,729	2%	ACTTCGGTTGCTGAAATTCT

Table S2. The sequences which were used as protospacers, TIDE primers and PCR primers for amplifying the inversion and wild-type junctions.

Protospacer Sequences	Sequence 5´-3´
RW65	ATTGTCTGGGCTAGATATGACTAG
RW66	AAACCTAGTCATATCTAGCCCAGA
RW67 (PS1)	ATTGCTTAGTATAAATAAGTCTGT
RW68 (PS2)	AAACACAGACTTATTTATACTAAG
RW69	ATTGAAGCAGCTACTGGGGTTAGC
RW70	AAACGCTAACCCCAGTAGCTGCTT
RW108 (PS2) Chr.3 fwd.	ATTGGCATGGCCGTGAGATTGAGC
RW109 (PS2) Chr.3 rev.	AAACGCTCAATCTCACGGCCATGC
RW110	ATTG TGAAATGAACCAAACAAAAC
RW111	AAACGTTTTGTTTGGTTCATTTCA
RW185 (PS3) Chr.3 fwd.	ATTGCTTCATGGATGTTTTATCAG
RW186 (PS3) Chr.3 rev.	AAACCTGATAAAACATCCATGAAG
RW187	ATTGCATGATTCAAGAAAATATAT
RW188	AAACATATATTTTCTTGAATCATG
RW189	ATTGACTTCGGTTGCTGAAATTCT
RW190	AAACAGAATTTCAGCAACCGAAGT

TIDE Primer Sequences

TIDE RW65/66 fwd.	CTACACAAGGGACTGTGTAG
TIDE RW65/66 rev.	CCCTCAAGCCCAGAATATGA
TIDE RW67/68 fwd.	GAGGGGATCGAGTATATGAG
TIDE RW67/68 rev.	ACCAGAGGTTCCACAACTAC
TIDE RW69/70 fwd.	CTACACAAGGGACTGTGTAG
TIDE RW69/70 rev.	GAATAGACACCCACCTAGTC
TIDE RW108/109 fwd.	GAAAGAGATGTTCCTGTG
TIDE RW108/109 rev.	GGGTACAGTTAAGAAAACC
TIDE RW110/111 fwd.	GACGCCGTTTGTGAAAAG
TIDE RW110/111 rev.	CCCCATTGATGTGAACGAG
TIDE RW185/186 fwd.	AAGAAGATAACAAATGGAGC
TIDE RW185/186, rev.	GCTAATCCTAATGGAATTTT
TIDE RW187/188 fwd.	GCCATGAATAGAATAGGTTG
TIDE RW187/188 rev.	TCACAATGATGTGTGAGCTC
TIDE RW189/190 fwd.	AAGAAGATAACAAATGGAGC
TIDE RW189/190 rev.	TCACAATGATGTGTGAGCTC

PCR Primers for Junctions	
Pericentomeric Inv. Ch.3 J1 fwd.	AACAAATGGAGCAGCCTCGAAC
Pericentomeric Inv. Ch.3 J1 rev.	TCTCCTAAACCTCAGTCGATTTCT
Pericentomeric Inv. Ch.3 J2 fwd.	CGAAGTATCCGAACCAATAACG
Pericentomeric Inv. Ch.3 J2 rev.	AACTACTCTCTCCCATCCTC
Pericentric Inv. Chr.3 J1 fwd.	TTGAGTCGAATCTCTTGATC
Pericentric Inv. Chr.3 J1 rev.	GCTATGTTGTTGTGATACAC
Pericentric Inv. Chr.3 J2 fwd.	AGGTTCCACAACTACTCTCT
Pericentric Inv. Chr.3 J2 rev.	GTGAGGACTTCCAACGAAAT

Table S3. Identified DEGs within the inverted regions of RW290, RW295 and CS1282. Out of the total number of 139, 327 and 167 DEGS detected in RW290, RW295 and CS1282, a small number of 4, 38 and 1 DEGs were located within the inversion segment and the flanking regions (± 100kb). The GO terms of the identified genes show that they are mostly involved in the regulation of metabolic and defense pathways.

Line	Total DEGs	No. of DEGs in inversion segment	Gene IDs	log_2FC	GO-Biological processes
RW290	139	4	AT3G23440	2,22	embryo development ending in seed dormancy
			AT3G24300	-2.09	lateral root formation; ammonium transmembrane transport
			AT3G27473	4,64	intracellular signal transduction
			AT3G29780	-2,23	calcium-mediated signaling
RW295	327	38	AT3G33035	5,22	-
			AT3G33084	4,89	DNA integration
			AT3G44020	2,27	-
			AT3G44326	2,46	regulation of steroid metabolic process
			AT3G44480	2,03	defense response to fungus
			AT3G44720	2,20	defense response
			AT3G44870	3,84	methylation
			AT3G45140	2,03	fatty acid biosynthetic process; response to herbivore
			AT3G45710	2,02	cellular response to salt
			AT3G46670	2,12	-
			AT3G47430	2,32	regulation of peroxisome size
			AT3G47950	-2,08	proton export across plasma membrane
			AT3G48320	2,50	-
			AT3G48350	2,22	proteolysis involved in cellular protein catabolic process
			AT3G48790	-2,47	sphingosine biosynthetic process
			AT3G50440	2,03	cellular response to hypoxia
			AT3G51070	4,64	methylation
			AT3G51220	2,01	-
			AT3G52561	-4,34	-
			AT3G53200	-2,22	response to chitin
			AT3G53680	2,30	positive regulation of transcription by RNA polymerase II
			AT3G53830	2,37	-
			AT3G53960	2,34	oligopeptide transport
			AT3G54420	2,25	defense response
			AT3G55500	-2,19	plant-type cell wall organization
			AT3G55710	2,14	
			AT3G56040	2,23	sulfolipid biosynthetic process
			AT3G56780	-2,10	-
CS1282	167	1	AT4G02715	2,10	-
